# Efficacy of topical minoxidil in enhancing beard growth in a group of transgender assigned female at birth individuals on gender affirming hormone therapy

**DOI:** 10.1007/s40618-024-02373-8

**Published:** 2024-04-21

**Authors:** L. Marinelli, A. Bichiri, S. Cagnina, L. Castella, E. Ghigo, G. Motta

**Affiliations:** Division of Endocrinology, Diabetes, and Metabolism, Department of Medical Sciences, University of Turin, 10126 Turin, Italy

**Keywords:** Transgender men, Beard, Hair growth, Minoxidil, Testosterone, AFAB individuals

## Abstract

**Purpose:**

Testosterone therapy represents the cornerstone of gender affirming hormone therapy (GAHT) among t-AFAB (transgender Assigned Female At Birth) people. Minoxidil is a vasodilator drug approved for topical use for the treatment of androgenetic alopecia. The aim of the present study was to evaluate the efficacy of topical minoxidil in enhancing beard growth in a group of t-AFAB people on GAHT.

**Methods:**

Sixteen t-AFAB individuals with an incomplete beard development, on GAHT for at least 6 months, were enrolled. Topical minoxidil was applied to the interested facial areas. Before starting (T0), after 3 (T3) and 6 (T6) months, we evaluated facial hair growth using the Ferriman-Gallwey modified score (FGm).

**Results:**

Subjects were 26 (2.7) years old and on GAHT for 18.5 [15–54] months; using a paired match evaluation, a statistically significant facial hair growth was observed over time, in particular at T6 (median upper lip FGm 3.5 [3–4] vs 2 [1–2] at T0 and chin FGm 4 [3.25–4] vs 1 [1–2] at T0; p ≤ 0.002). Comparing the minoxidil group with a control group (n = 16) matched for age and BMI who developed a full-grown beard only with GAHT, a logistic multivariable analysis identified hirsutism before GAHT was independently positively associated with the development of a full beard [OR 15.22 (95% CI 1.46–158.82); p = 0.023].

**Conclusions:**

This is the first study demonstrating the efficacy of topical minoxidil in enhancing facial hair growth among t-AFAB people on GAHT. Further studies will be necessary to assess whether the obtained improvements will persist after discontinuing the medication.

## Introduction

Gender Incongruence relates to a marked and persistent incongruence between an individual’s experienced gender and the assigned gender at birth [1]. To align a person’s body with their gender identity, transgender and gender diverse (TGD) people may ask for gender-affirming medical and surgical treatments (GAMSTs) including gender-affirming hormone therapy (GAHT) and gender-affirming surgery (GAS) [2].

GAHT in TGD assigned female at birth (t-AFAB) individuals aims to obtain a certain degree of masculinization and/or defeminization. In this context, the cornerstone is represented by testosterone therapy. Different testosterone formulations are available, as endorsed by the 8th version of the Standards of Care for the Health of Transgender and Gender Diverse People published in 2022 [2]; these include transdermal formulations and parenteral injections such as short-term testosterone esters (enanthate or mixed esthers) or long-acting undecanoate testosterone [3]. The masculinizing effects related to testosterone use are numerous; some of them include increased facial and body hair, heightened lean mass and strength, deepening of the voice, amenorrhea, clitoral enlargement and an increased incidence of acne and androgenetic alopecia [4–8]. However testosterone may not always be sufficient to promote a full facial hair development and some t-AFAB individuals who cannot attain the desired virilization may experience significant distress [9].

Minoxidil is a vasodilator oral drug initially approved by the FDA in 1979 to treat refractory hypertension [10]. Coincidentally, generalized hypertrichosis in balding patients was observed as an adverse effect [11], which led to the development of a topical formulation to treat both androgenetic alopecia and female pattern hair loss. Its use in these dermatological conditions represents the ongoing on-label indications; nonetheless, minoxidil is widely used in off-label regimens for other hair loss conditions including alopecia areata, scarring alopecia and hair shaft disorders. Additionally, it is used to improve body hair growth in other areas, such as the face [12]. Minoxidil mechanisms of action are still not fully elucidated, but it has been shown to induce a strong arteriolar vasodilator effect mediated by the opening of K^+^ channels on smooth muscle cells of peripheral arteries [13, 14].

The potential use of minoxidil to stimulate hair growth or regrowth could extend to beard enhancement. To support this finding, only two studies are currently available in the literature. The first one is a randomized, double-masked, placebo-controlled study published in 2016 that enrolled 48 cisgender men (20–60 years old) desiring a more complete facial hair development [15]. Following a 16 week treatment period, Ingprasert et al. demonstrated the efficacy of minoxidil 3% lotion (applied 0.5 ml twice daily) versus placebo based on global photographic scores, mean change from baseline in hair count and patients’ self-assessments. Among the TGD population there is only one case-report of a 17 year-old t-AFAB adolescent who was initially unable to access GAHT and requested minoxidil to increase facial hair and reduce misgendering together with gender dysphoria [16]. Pang et al. used the modified Ferriman Gallwey (FGm) score to monitor the patient and observed an increase in beard growth after 3 months of minoxidil (5% lotion) monotherapy (upper lip FGm score from 0 to 2, and chin FGm score from 0 to 3).

After these premises, the present study aimed to investigate the efficacy of topical minoxidil in a group of t-AFAB individuals on GAHT who did not develop a full beard.

## Materials and methods

We conducted a prospective interventional study, which included t-AFAB individuals who were followed up by our gender team at “Città della Salute e della Scienza” University Hospital in Turin, Italy.

The study design was divided into two steps. To be included in the first phase of the study, t-AFAB people had to fulfil the following inclusion criteria:

- to be on GAHT with only testosterone for a minimum of 6 months;
- not to have developed a fully-grown beard (defined as a facial FGm score less than 4 at both the chin and the upper lip);
- expressed desire to enhance beard growth;
- not to have used any formulation of minoxidil before the time of enrollment;
- not to take any other drugs or supplements, to prevent any interference with the results.

These enrolled subjects were labeled as the “minoxidil group”.

Before starting GAHT, the following information was collected: age, Body Mass Index (BMI), menstrual history, ovaries ultrasound, clinical hyperandrogenism (in particular hirsutism, defined as a total FGm ≥ 8) and biochemical hyperandrogenism [defined as at least one of the following androgens above the upper reference limit [total testosterone (TT), 17-hydroxyprogesterone, (17OHP), dehydroepiandrosterone sulphate (DHEA-s), Δ-4-androstenedione)].

At T0, a topical preparation of minoxidil 2% lotion, 2 ml once daily was used in a group of patients who met the inclusion criteria. People were carefully instructed on how to apply the lotion, specifically 1 ml to each half of the face, distributing equally on the interested facial areas (upper lip, chin, upper and lower jaw). Finally, we documented the following parameters: age, BMI, months of GAHT, typology of testosterone preparations (transdermal, intramuscular long- or short-acting), compliance to GAHT, (a medication possession ratio of ≥ 80% was considered as a marker of “good compliance”), time to the induction of amenorrhea (weeks) and TT and estradiol (E2) serum levels.

After 3 (T3) and 6 months (T6), facial hair growth development was reassessed using the FGm score, in order to evaluate potential modifications. To reduce variability, the hair assessment was performed by the same clinician each time.

All hormonal evaluations were obtained by venous blood samples between 8 and 10 am after an overnight fasting.

In the second phase of the study, we conducted a comparison between the “minoxidil group” and a control group of t-AFAB individuals who met the following inclusion criteria:

- to be on GAHT with only testosterone for a minimum of 6 months;
- to have developed a fully-grown beard (defined as a facial FGm score of 4 at both the chin and the upper lip);
- not to have used any formulation of minoxidil before the time of enrollment;
- not to have taken any other drugs or supplements with a potential influence on hair growth.

The comparison included data collected before GAHT and at the time of enrollment.

**Fig. 1:**
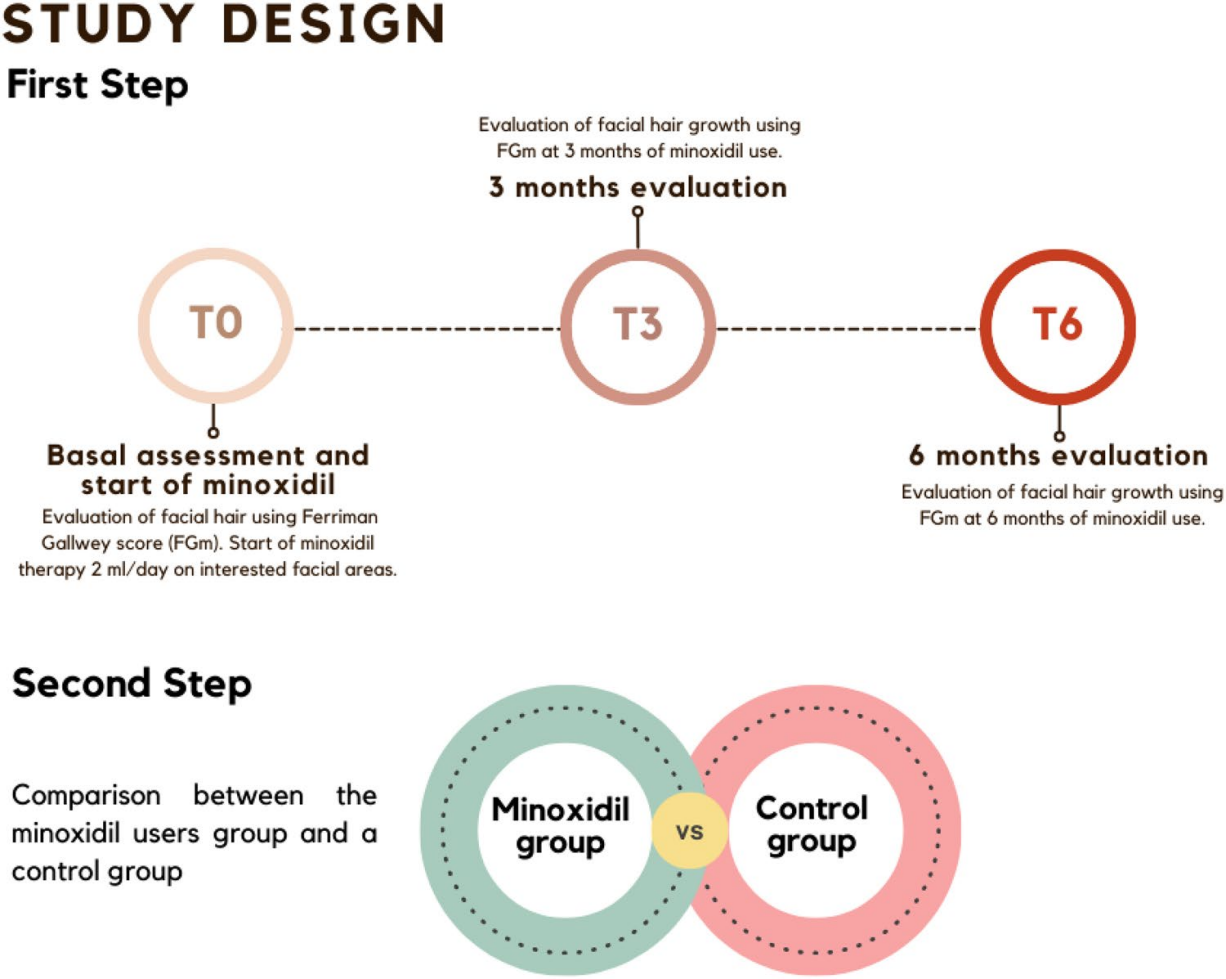
Study design

A graphical representation of the study steps is available in Fig. 1.

Written informed consent was obtained from all patients, with the approval of the local Ethical Committee (number 0032354 Sept. 25th, 2012).

### Statistical analysis

Continuous skewed variables are presented as median [25th–75th percentiles] while normally distributed data as mean (SD). Categorical variables were reported as relative frequencies (%). Friedman’s ANOVA was conducted to identify any differences in variables between T0, T3, and T6. To confirm significant differences over time, Wilcoxon tests were performed, and Bonferroni post hoc corrections were applied to control the risk of Type I errors due to multiple comparisons.

Comparisons between the minoxidil and full beard groups were conducted as follows: the Mann–Whitney U test was used for skewed data, Student's t-test was used for normally distributed data, while Fisher’s exact test was used for categorical variables. Bonferroni post hoc analyses were performed as multiplicity adjustment procedures. Logistic multivariable analyses were conducted adopting a stepwise regression approach.

The statistical analysis was conducted using the IBM SPSS program (IBM SPSS Statistics for Windows, Version 28.0. Armonk, NY, USA: IBM Corp). The statistical significance level was set at p < 0.05.

## Results

Sixteen t-AFAB individuals were enrolled in the first phase of the study (the “minoxidil group”). The baseline characteristics of this group before starting GAHT are summarized in Table 1. Only 13% reported a clinically significant hirsutism, 12% of the sample experienced oligo-amenorrhea, and no cases of polycystic ovary morphology were described in this sample.

**Table 1:** Characteristics of the minoxidil users (minoxidil group) before starting GAHT

Transgender AFAB individuals (n = 16)	
Before starting GAHT	
Age (years)	23.44 (4.10)
BMI (kg/m^2^)	23.61 (5.21)
Total testosterone (ng/dL)	45 (21)
17-OHP (ng/mL)	1.13 [0.64–1.60]
DHEA-s (µg/dL)	2437.54 (879.23)
Androstenedione (ng/dL)	2.70 (1.29)
PCOM (%)	0
Hirsutism before starting GAHT (%)	13.33
Menstrual cycle before starting GAHT	87.5 eumenorrhea
	12.5 oligomenorrhea

*AFAB* assigned Female at Birth, *GAHT* gender affirming hormone therapy, *BMI* body mass index, *17-OHP* 17-hydroxyprogesterone, *DHEA-s* dehydroepiandrosterone-sulphfate, *PCOM* polycystic ovary morphology

At T0, before starting minoxidil, the mean age of the enrolled t-AFAB individuals was 26 (2.7), and they were on GAHT for a median of 18.5 [15–54] months using different testosterone formulations, although the majority (68%) was on long-acting intramuscular testosterone undecanoate injections. All reported a good compliance with GAHT, as reflected by an average serum testosterone concentration of 565 (267) ng/dL. In terms of beard development, a median FGm score of 2 [1, 2] at the upper lip and of 1 [1, 2] at the chin were observed at T0 (Table 2).

**Table 2:** Characteristics of the minoxidil users (minoxidil group) before starting minoxidil (T0)

Transgender AFAB individuals (n = 16)	
Before starting minoxidil (T0)	
Age (years)	26.12 (2.85)
BMI (kg/m^2^)	24.06 (4.86)
Total period of GAHT (months)	18.5 [15–54]
Time to amenorrhea (weeks)	16 [4–30]
Type of testosterone therapy (%)	69 long-acting IM
	25 transdermal
	6 short-acting IM
Good compliance to GAHT (%)	100
Total testosterone (ng/dL)	565 (267)
Estradiol (pg/mL)	50 [34–110.10]
FGm upper lip	2 [1–2]
FGm chin	1 [1–2]

*AFAB* assigned female at birth, *BMI* body mass index, *GAHT* gender affirming hormone therapy, *IM* intramuscularly, *FGm* modified Ferriman-Gallwey score

At T3 only 15 t-AFAB individuals remained on therapy, as one participant discontinued minoxidil after 1 week due to flushing and skin irritation. We observed a median upper lip FGm score of 3 [2.25–4] and chin of 3.5[3, 4]. At T6, 12 t-AFAB individuals were still using minoxidil, while three decided to stop the treatment due to personal satisfaction with the obtained results. The median FGm scores were 3.5 [1, 2] at the upper lip and 4 [3.25–4] at the chin.

Considering the overall changes over time, the Friedman test for non-parametric paired data showed a significant increase in hair growth for both the chin [χ ^2^(2) = 17.70, p < 0.001] and the upper lip [χ ^2^(2) = 17.68, p < 0.001] during observation. A post hoc analysis with Wilcoxon signed-rank tests was conducted with a Bonferroni correction applied, to compensate for multiple comparisons; this resulted in a significance level set at *p* < 0.008. Specifically, a noteworthy increase was observed from T0 to T3 for both chin (z = 2.85; p = 0.004) and upper lip (z = 3.03; p = 0.002) and from T0 to T6 (chin: z = 3.21; p = 0.001; upper lip: z = 3.10; p = 0.002). No significant differences were detected between T3 and T6 for both sites (chin: z = 2.12; p = 0.03, upper lip: z = 1.63; p = 0.10). Table 3 summarize the progression of FGm scores over time.

**Table 3:** Progression of facial hair growth after starting minoxidil (T0)

	T0 (n = 16)	T3 (n = 15)	T6 (n = 12)	Paired T0 vs T3	Paired T0 vs T6	Paired T3 vs T6
FGm upper lip	2 [1, 2]	3 [2.25–4]	3.5 [3, 4]	p = 0.004	p = 0.001	NS
FGm chin	1 [1, 2]	3.5 [3, 4]	4 [3.25–4]	p = 0.002	p = 0.002	NS

Statistical significance was set at p < 0.008 after adjustment for post-hoc analysis

*T*0 before starting minoxidil, *T*3 after 3 months of treatment with minoxidil, *T*6 after 6 months of treatment with minoxidil, *FGm* Ferriman-Gallwey modified score

The users tolerated the topical therapy well, and no major side effects related to the minoxidil use were reported. However, among minor side effects, one person complained of flushing and skin irritation that led to discontinuation of the topical therapy.

In the second phase of the study, we compared the “minoxidil group” with a “control group” matched for age and BMI. Differences among studied variables were compared and a post hoc analysis with a Bonferroni correction was used to reduce type I error. No significant differences were found between the two groups in any of the considered variables (Table 4).

**Table 4:** Comparisons between the minoxidil group and the control group

	Minoxidil group (n = 16)	Control group (n = 16)	p
Age before starting GAHT (years)	23.44 (4.10)	24.81 (5.88)	NS
BMI before starting GAHT (Kg/m^2^)	23.15 [20.1–26]	22.5 [20.5–26.7]	NS
Total testosterone before starting GAHT (ng/dL)	45 (21)	43 (17)	NS
17-OHP before starting GAHT (ng/mL)	1.13 [0.64–1.60]	0.97 [0.80–1.71]	NS
DHEA-s before starting GAHT (µg/dL)	2437.54 (879.23)	2462 (1086.30)	NS
Androstenedione before starting GAHT (ng/dL)	2.70 (1.29)	2.80 (1.17)	NS
Hirsutism before starting GAHT (%)	13.33	46.67	NS
PCOM (%)	0	6.67	NS
Menstrual cyclicity before starting GAHT (%)	87.50 eumenorrhea	81.25 eumenorrhea	NS
	12.5 oligomenorrhea	18.75 oligomenorrhea	NS
Total period of GAHT (months)	18.50 [15–54]	35 [24–57]	NS
Amenorrhea after GAHT (weeks)	16 [4–30]	6 [4–17.5]	NS
Typology of testosterone therapy (%)	69 long-acting IM	87.5 long-acting IM	NS
	25 transdermal	12.5 transdermal	NS
	6 short-acting IM		
Good compliance to GAHT (%)	100	100	NS
Age at the enrollment (years)	26.12 (2.85)	28.06 (6.70)	NS
Testosterone serum levels at enrollment (ng/dL)	565 (2.67)	573 (200)	NS
Estradiol serum levels at enrollment (pg/mL)	50 [34–110]	45 [34–58]	NS

Statistical significance was set at p < 0.005 after adjustment for post-hoc analysis

*GAHT* gender affirming hormone therapy, *BMI* body mass index, *17-OHP* 17-hydroxyprogesterone, *DHEA-s* dehydroepiandrosterone-sulphfate, *PCOM* polycystic ovary morphology, *IM* intramuscularly

Analysing the two groups altogether, a logistic regression was performed to ascertain the effects of the presence of hirsutism and TT serum levels before GAHT on the likelihood of obtaining full facial hair. The logistic regression model was statistically significant, χ ^2^(1) = 7.363, *p* = 0.025. The model accounted for 34.0% (Nagelkerke *R*^*2*^) of the variance in full beard development and correctly classified 63.0% of the cases. The presence of hirsutism before starting GAHT increased the likelihood of developing a full beard by 15 times [OR 15.22 (95% CI 1.46–158.82); p = 0.023] (Table 5).

**Table 5:** Multivariable logistic regression for obtaining a full beard growth

	OR	95% CI	p
Hirsutism	15.22	1.46–158.82	0.023
Serum total testosterone before GAHT	0.02	0.00–3.79	0.14

Pseudo R-Squared: 0.34, p-value model: 0.025. Statistical significance was set at p < 0.05

*OR* Odds Ratio, *CI 95%* Confidence Interval, *GAHT* gender affirming hormone therapy

## Discussion

To the best of our knowledge, this is the first prospective interventional clinical study estimating the impact of minoxidil 2% off-label for beard enhancement in a group of t-AFAB individuals on GAHT.

Within our cohort, the application of topical minoxidil 2% proved to be effective in improving facial hair growth after 3 and 6 months, at both the chin and the upper lip, compared to baseline assessments. Notably, it has to be considered that these effects occurred in a relatively short period of time. Hairs grow in nonsynchronous cycles, and the growth phase (anagen) is known to last 4 months for facial hair [17]. Surprisingly, in our cohort a significant improvement in beard development was already evident at 3 months. This may be explained by a previous activation of the dermal papilla triggered by elevated androgen serum levels: in fact, testosterone has been shown to increase the size of the dermal papilla and consequently the hair size in both cisgender men and women [18]. This mechanism may have boosted the effect of minoxidil on facial hair growth.

T-AFAB individuals on GAHT with testosterone can experience a different nuance of virilization. The factors involved in this variety of phenotypes are not yet fully grasped. One of the pivotal determinants highlighted by researchers is the time on testosterone therapy. In fact, GAHT duration represent a factor that impacts on virilization, due to time-dependent effects of testosterone starting after three months of use [19]. In our cohort, however, the extended range of time on GAHT (18.5 [15–54] months), demonstrates that even individuals with longer time on testosterone may suffer from an incomplete facial virilization. In this scenario, minoxidil showed to be efficacious in improving beard growth. On the other hand, using different testosterone formulations has shown not to influence virilization, regardless of achieving similar serum testosterone levels and safety profile [20–23].

Despite similar preliminary results regarding the efficacy of minoxidil on beard growth reported by Ingprasert et al., that study design was quite different from the present one: the authors enrolled cisgender men with unknown serum testosterone levels and used an evaluation protocol based on a photographic score [15]. The present study represents the first report on the efficacy of minoxidil in beard development within a t-AFAB population in addition to testosterone therapy. Thus, minoxidil may represent an adjunctive therapeutic tool for t-AFAB individuals seeking complete facial virilization during testosterone therapy.

No peculiar side effects related to minoxidil use were reported in our population. Only one individual experienced early side effects (flushing and skin irritation) that led to therapy discontinuation, while the others well tolerated the drug. Despite the low incidence, literature reports the possibility to incur into contact dermatitis, pruritus, and skin irritation during minoxidil use for alopecia treatment [24, 25]. Consequently, t-AFAB users should be properly informed before minoxidil prescription.

To better understand the effect of minoxidil independent of testosterone therapy in t-AFAB individuals, in the second phase of the study we compared the minoxidil group with a control group of t-AFAB people on GAHT who developed a full-grown beard without employing additional drugs.

Differently from what one might think, in the control group basal serum androgens levels were not significantly higher and clinical characteristics were not significantly diverse when compared to the minoxidil one. Furthermore, a logistic regression model showed that in our cohort the presence of hirsutism before starting GAHT was independently associated with the development of full beard [OR 15.22 (95% CI 1.46–158.82)], confirming that basal hirsutism is a crucial predisposing factor for beard development. Hirsutism is defined as an excessive terminal hair growth that appears in a male pattern in cisgender women [26]. Although androgen excess underlies most cases of hirsutism, hirsutism scores do not properly correlate with detectable androgen levels [27, 28]. This can be explained by the fact that stimulation of hair growth does not solely depend on circulating androgen concentrations. Local factors and the variability of the androgen-dependent pilosebaceous follicle response also play a role [17]; these mechanisms result in the activation of the pilosebaceous unit, leading to the transformation of the vellus in terminal hair [17]. Eventually, these interactions may explain why in our study enrolled t-AFAB individuals with baseline hirsutism experienced a greater  improvement on facial hair growth using minoxidil.

For the reasons abovementioned, routinely assessed serum androgens (e.g., TT, DHEA-s, androstenedione, 17OHP) may not always be elevated in hyperandrogenic conditions, such as hirsutism and androgenetic alopecia. Thus, an extensive evaluation of the androgen metabolites, using mass spectrometry, could be helpful in the future for a more precise differential diagnosis of these idiopathic cases [29] and potentially to unveil new therapeutic targets. In greater detail, no differences between the two groups were also highlighted in terms of serum testosterone levels during GAHT, the typology of testosterone preparations or the total duration of GAHT.

Minoxidil seems to embody a valuable and versatile tool in everyday clinical practice in transgender care and it may be proposed in two different clinical settings. First, in t-AFAB individuals on GAHT to enhance beard growth, as demonstrated by our prospective study. Second, in non-binary t-AFAB individuals who do not request GAHT [30]. The number of non-binary t-AFAB people who do not ask for GAHT or do request testosterone microdosing is, in fact, increasing [31]. These individuals may attempt a partial virilization to avoid specific effects of testosterone use, such as the deepening of the voice or the potential fertility impairment [32]. Minoxidil can represent a helpful drug in this context. No less importantly, as demonstrated in a case report by Pang. et al [16], minoxidil can be a valid aid to reduce misgendering when a t-AFAB person is unable to promptly access GAMSTs.

## Limitations

Due to the nature of the study, our results cannot be generalized for non-medicalized TGD population. GAHT lacked standardization across the study participants due to variability in testosterone formulations and time of administration. Furthermore, the FGm score employed in our study represents a tool validated in cisgender women affected by hirsutism while a specific one to evaluate facial hair growth in TGD population does not currently exist. To improve the robustness of our results a greater number of enrolled people should be included.

## Conclusion

This study demonstrated how topical 2% minoxidil was efficacious and not harmful to promote facial hair growth in a group of t-AFAB individuals on GAHT. Minoxidil may represent a valuable tool in everyday clinical practice among t-AFAB people on GAHT who desire to enhance beard growth.

Further studies will be necessary to assess whether discontinuing the drug results in maintaining the obtained improvements and whether among TGD people who do not desire testosterone therapy, topical minoxidil will be sufficient to steadily develop facial hair.

## Data Availability

The datasets generated during and/or analysed during the current study are available from the corresponding author upon reasonable request.
